# Analysis of the Clinical Outcome of Arthrographic Steroid Injection for the Treatment of Adhesive Capsulitis

**DOI:** 10.2174/1874325001711010804

**Published:** 2017-08-25

**Authors:** Allison Tucker, Hiscox Christina, AlQahtani Saad, Ryan Bicknell

**Affiliations:** Department of Surgery, Kingston Health Sciences Center and Queen’s University, Kingston, Ontario, Canada

**Keywords:** Adhesive capsulitis, Frozen shoulder, Steroid, Local anesthetic, Injection, Arthrogram

## Abstract

**Background:**

Idiopathic adhesive capsulitis is a condition of uncertain etiology characterized by pain and decreased shoulder range of motion (ROM) that occurs without a known intrinsic disorder. Many treatments have been advocated, yet the best option remains unclear. The purpose of this study was to determine if arthrographic injection of the shoulder joint with steroid and local anesthetic results in decreased pain and increased shoulder function in a cohort of patients with idiopathic adhesive capsulitis.

**Methods:**

This is a retrospective case series of patients who were treated with an arthrographic steroid and local anesthetic injection for adhesive capsulitis. The injections were all given by the same musculoskeletal radiologist using image guidance, and the patients were all from the same surgeon's practice. Patients were evaluated with the Shoulder Pain and Disability Questionnaire (SPADI) and Constant Shoulder Score and their shoulder ROM was tested. Descriptive statistics in the form of counts, percentages, means and standard deviations were used, as well as parametric and non-parametric tests.

**Results:**

Thirty-three shoulders in 25 patients were examined. The average length of follow-up was 17 months. Sixty-nine percent of the cohort continued to complain of some shoulder pain in the post-injection follow-up clinic, however, their pain had improved from 8.8/10 pre-injection to 2.2/10 post-injection (p=0.01). The average score of the SPADI Pain, SPADI Disability and Constant Score were 36, 31 and 64, respectively.

**Conclusion:**

All patients who received an arthrographic injection using steroid and local anesthetic reported improved pain and mobility. In addition, those who had tried other treatment modalities felt that the injection had been the most beneficial.

## 1 INTRODUCTION

Frozen shoulder, or adhesive capsulitis, is a common cause of shoulder pain that is estimated to affect 2–5% of the general population [1-3]. The condition was first described by Duplay in 1872 with the term “frozen shoulder” coined by Codman in 1934 [2, 4-7]. More recently, Zuckerman and Cuomo defined frozen shoulder, or idiopathic adhesive capsulitis, as a condition of uncertain etiology characterized by substantial restriction of both active and passive range of motion that occurs in the absence of a known intrinsic shoulder disorder [2, 8].

Initially, frozen shoulder was felt by some to be a self-limiting condition with a favorable natural history [3, 7]. However, other studies reported up to 40% of patients continued to have limited motion and pain 3 years after diagnosis [9, 10]. A study by Shaffer *et al*. showed that 50% of patients treated non-operatively had continued mild pain or stiffness or both [2, 11].

Many forms of treatment have been advocated for frozen shoulder including physiotherapy, injection with steroid, distension arthrogram, manipulation under anesthetic and arthoscopic releases [1, 3, 6, 12, 13]. Despite the vast armamentarium of treatment options available, there continues to be no general consensus on the optimal treatment regime for this patient population [6, 14].

One randomized control trial evaluated the use of an intra-articular corticosteroid injection in a diabetic population with adhesive capsulitis. The researchers found that those patients treated with the injection had less pain at 6 weeks, better function at 12 weeks, but no lasting differences when compared with the control group who did not receive an injection [15]. Buchbinder *et al*. randomly assigned patients diagnosed with adhesive capsulitis to receive either arthrographic joint distension with steroid and saline or a placebo consisting of simply the arthrogram. They found that patients receiving the distension arthrogram with steroid and saline had significant improvements at 3 weeks in their SPADI, PET, overall pain, active abduction and internal rotation with their hand behind their back [16]. A recently published meta-analysis reported intra-articular steroid injections to be safe and effective for frozen shoulder at short-term follow-up [17]. As the authors point out, however, only 1 of the 8 included randomized controlled trials were free from bias.

The objective of this retrospective study was to determine if arthrographic injection of the shoulder joint with steroid in the mid tern resulted in decreased pain and increased shoulder function in a cohort of patients who presented with adhesive capsulitis.

## 2 METHODS

This is a retrospective case series study of patients who had been treated with an image-guided arthrographic injection using steroid for adhesive capsulitis in one surgeon’s practice. The inclusion criteria comprised patients between 18-70 years of age with a diagnosis of primary adhesive capsulitis that was treated with an image-guided arthrographic injection using 40 mg of Triamcinilone and 5cc of 1% Lidocaine by the same musculoskeletal radiologist. The minimum follow-up was 3 months. The exclusion criteria included patients who developed adhesive capsulitis secondary to trauma or who had had a previous surgery on the affected shoulder.

Patients were evaluated with questionnaires and their range of shoulder motion tested with a goniometer. Questionnaires included the SPADI (Shoulder Pain and Disability Index) and the Constant Score Shoulder questionnaire. The SPADI is a validated, self-report measure developed to evaluate patients with shoulder pathology. It contains two domains; one is a 5-item subscale that measures pain and the other an 8-item subscale that measures disability. The pain dimension has five questions regarding the severity of an individual’s pain and the functional activities dimension has eight questions designed to measure the degree of difficulty a patient has while carrying out the activities of daily living. The scores from both dimensions are averaged to derive a total score with 0 being the best outcome (less disability) and 100 the worst (greater disability) [18].

The Constant Score is also a validated instrument used to assess pain and function of a diseased shoulder. It has a maximum score of 100: 15 points are allocated for a subjective assessment of pain, 20 points for shoulder function, 40 points for an objective measure of ROM and 25 points for shoulder strength. A higher score illustrates better function [19]. Subjective pain was also recorded using a visual analog scale of 0 (no pain) - 10 (severe pain). It should be noted that all patients were encouraged to try physiotherapy at their index appointment prior to their injection. This study was reviewed and approved by our institution’s Research Ethics Board.

Statistical analysis included descriptive statistics in the form of counts, percentages, means and standard deviations to summarize demographic and other study variables. Parametric and non-parametric tests were used to compare variables between sexes, as well as pre and post injection differences when possible. An alpha value <.05 was considered statistically significant.

## 3 RESULTS

A total of 33 shoulders in 25 patients were enrolled in the study. Eighteen of the participants were female and 7 were male with an average age of 57 years and a range of 47-70 years. Twenty-six of the shoulders examined were right with 7 left and 83% of the cohort were right hand dominant. The average follow-up was 17 months post-injection with a range of 4-41 months. Multiple co-morbidities were identified in the cohort. The most common were diabetes, pulmonary disease and heart disease at 44%, 21% and 18% respectively. Please refer to (Table **1**) for a summary of patient characteristics.

The average number of injections per shoulder in the cohort was 1.4. There was a range of 1-3 injections. Eighteen patients had one injection while 6 patients had 2 and 1 patient had 3 injections. Patients reported trying other treatment modalities including medication specifically for their shoulder symptoms (87%), physiotherapy (67%), massage therapy (47%) and acupuncture (17%). For those who tried physiotherapy and massage, the average number of sessions were 27 and 5 respectively. The patients taking medications mostly took Tylenol or aspirin daily.

Of the 6 patients that received 2 injections, 3 were men and 3 were women. All were right hand dominant expect 1. Half of these patients were diabetic and all were taking insulin; one was a smoker. They all reported taking weekly medication for the pain with minimal improvement. The one patient that had 3 injections was also diabetic and on insulin; was right hand dominant and was taking Tylenol for the pain with no relief. The average pain severity score of these 7 patients was 9.5

Sixty-nine percent of the cohort continued to complain of some shoulder pain in the post-injection follow-up clinic, however, their pain had improved from a mean of 8.8/10 pre-injection to 2.2/10 post-injection (p=0.01). The average score of the SPADI Pain was 36/100 and the SPADI Disability was 31/100 for a total of 67/100. This is within the normative average of 61/100. The Constant Score total was 64/100 which is below the normal average of approximately 84 for this age group. Range of motion measures included forward flexion which was 130.5° (opposite=139.8), abduction was 120.2° (opposite=142.5), and based on the Constant scoring system of 0-10, external rotation was 8 and internal rotation was 4.3 for the index side (opposite= 9 on average for both). No patients reported any increased pain with the injection or within 24 hours post-injection and there were no other reported complications. Furthermore, there were no statistically significant differences on any measures between the men and women in our cohort.

## DISCUSSION

We found injections with steroid increased shoulder motion and function, and significantly decreased pain in our cohort, although not entirely. This same finding was reported in a recently published systematic review examining intra-articular steroid injection for frozen shoulder [17]. The optimal treatment regime for adhesive capsulitis remains unclear [6, 14]. According to one review paper, all treatment options for this condition resulted in improved clinical outcomes as measured by the Constant, SPADI and VAS pain scores [20]. Interestingly, intra-articular injection with steroid was found superior in the short-term when compared to the other treatment modalities including physiotherapy, manipulation under anesthetic, hydraulic joint distention and oral steroid [1, 14, 20, 21].

Carette *et al*. produced similar results finding that patients treated with intra-articular steroid injection had improved pain and function scores than those treated with a saline injection alone or in combination with supervised physiotherapy. They did however identify that those patients who received a combination of an intra-articular steroid injection and supervised physiotherapy program had the fastest improvement in their range of motion [22]. These finding were later reported by Blanchard, Barr and Cerisola in 2009 who completed a systematic review of the literature. Their review suggested that corticosteroid injection produced more benefits when compared to physiotherapeutic intervention in patients with adhesive capsulitis, at least in the short-term [1]. These findings are of course not surprising or novel as Bulgen *et al*. identified the short-term benefit of intra-articular steroid injection when compared with mobilization, ice and rest back in 1984 [14]. Looking at other forms of steroid, Lorbach *et al*. studied intra-articular steroid injection versus a course of oral steroid in the treatment of adhesive capsulitis [21]. They found that the patients who received the intra-articular steroid injection had improved pain control, range of motion and patient satisfaction compared to the group who received the course of oral steroid. This study was in keeping with the previous literature as 100% of the patients reported improved pain and mobility post arthrographic steroid injection. In addition, those who had tried other treatment modalities felt the injection had been the most beneficial.

The strengths of this study include that all patients were recruited from the same surgeon’s practice and thus had the same evaluation, received the same recommendations and were offered the same treatment options. In addition, all of the arthrographic steroid injections were performed by the same experienced musculoskeletal radiologist who used a standard technique with each patient receiving 40 mg of Triamcinilone and 5cc of 1% Lidocaine. There were no complications in our cohort such as infection, facial flushing or increased pain post-injection. This is consistent with the literature as arthrographic injection using steroid is considered a relatively benign and inexpensive procedure with few inherent risks to the patient [23].

This study had several limitations inherent to a retrospective study. The response rate was quite low; however, all patients were recruited from the same surgeon’s practice, which although limited our numbers, added to the consistency. The follow-up interval had a wide range from 4-41 months. Consequently, it would have been preferable to stratify the results but unfortunately, the small number of participants did not allow for this to be accomplished. In addition, the number of injections varied from 1-3 in the index shoulder of our participants, which could have influenced their response to the treatment. Finally, we did not have any pre-injection data for our subjects such as their range of motion, SPADI or Constant score. Subjectively, they all reported significant improvements in their level of pain, motion and functional abilities.

## CONCLUSION

Arthrographic injection with steroid is a safe treatment option for patients with adhesive capsulitis that appears beneficial in improving both pain and range of motion. Despite the limitations in our study, we feel arthrographic injection with steroid is a viable treatment option with very little downside. Thus, we continue to recommend it as a treatment option for patients with frozen shoulder.

Future directions include a randomized control trial in which we will compare arthrographic injection with steroid, local anesthetic and saline to injection with local anesthetic and saline alone to determine whether steroid is actually of benefit to this patient population.

## Figures and Tables

**Table 1:** Patient Characteristics.

Characteristic	No. of Shoulders (n=33)
Age	57 (47-70)
Sex: male/female	18/7
Side: right/left	26/7
Dominance: right/left	27/6
Average follow-up (months)	17 (4-41)
Co-morbidities (%)	
Diabetes Pulmonary disease Heart disease	44 21 18
